# Is it possible to diagnose therapeutic adherence in mild cognitive impairment and dementia patients in clinical practice?

**DOI:** 10.3389/fphar.2024.1362168

**Published:** 2024-05-22

**Authors:** Pilar Barnestein-Fonseca, Gloria Guerrero-Pertiñez, Jose Gúzman-Parra, Esperanza Valera-Moreno, Fermín Mayoral-Cleries

**Affiliations:** ^1^ Research Unit, Instituto CUDECA de Estudios e Investigación en Cuidados Paliativos, Fundación CUDECA, Málaga, Spain; ^2^ Instituto IBIMA-Plataforma Bionand, Málaga, Spain; ^3^ UGC Mental-Health, Hospital Regional de Málaga, Andalusian Health Service, Málaga, Spain

**Keywords:** cognitive impairment, mild dementia, treatment adherence, adherence indirect test, self-reported methods

## Abstract

**Background:**

Non-adherence is common and contributes to adverse health outcomes, reduced quality of life, and increased healthcare expenditure. The objective of this study was to assess the diagnostic validity to estimate the prevalence of non-adherence in patients with mild cognitive impairment (MCI) and dementia using two self-reported methods (SRMs) that are useful and easy in clinical practice, considering the pill count as a reference method (RM).

**Methods:**

The cohort study was nested in a multicenter randomized controlled trial NCT03325699. A total of 387 patients from 8 health centers were selected using a non-probabilistic consecutive sampling method. Inclusion criteria were as follows: a score of 20–28 points on the Mini-Mental State Examination (MMSE); older than 55 years; taking prescribed medication; and are in charge of their own medication use. Participants were followed up for 18 months after the baseline visit, i.e., 6, 12, and 18 months. Variables related with treatment adherences were measured in all visits. The variables included age, sex, treatment, comorbidities, and the MMSE test. Adherences included pill counts and Morisky–Green test (MGT) and Batalla test (BT) as SRMs. Statistical analysis included descriptive analysis and 95% confidence intervals (CIs). The diagnostic validity included the following: 1) open comparison statistical association between SRMs and RMs and 2) hierarchy comparison: the RM as the best method to assess non-adherence, kappa value (k), sensitivity (S), specificity (Sp), and likelihood ratio (PPV/PPN).

**Results:**

A total of 387 patients were recruited with an average age of 73.29 years (95% CI, 72.54–74.04), of which 59.5% were female. Comorbidities were 54.4% HTA, 35.9% osteoarticular pathology, and 24.5% DM. The MMSE mean score was 25.57 (95% CI, 25.34–25.8). The treatment adherence for the RM oscillates between 22.5% in the baseline and 26.3%, 14.8%, and 17.9% in the follow-up visits. For SRMs, the treatment adherence oscillates between 43.5% in the baseline and 32.4%, 21.9%, and 20.3% in the follow-up visits. The kappa value was statistically significant in all the comparison in all visits with a score between 0.16 and 035. Regarding the diagnostic validity, for the MGT, the sensibility oscillated between 0.4 and 0.58, and the specificity oscillated between 0.68 and 0.87; for the BT, the sensibility oscillated between 0.4 and 0.7, and the specificity oscillated between 0.66 and 0.9; and when both tests were used together, the sensibility oscillated between 0.22 and 0.4, and the specificity oscillated between 0.85 and 0.96.

**Conclusion:**

SRMs classify non-adherent subjects correctly. They are very easy to use and yield quick results in clinical practice, so SRMs would be used for the non-adherence diagnosis in patients with MCI and mild dementia.

## 1 Introduction

The global demographic landscape is undergoing a profound shift, marked by an undeniable surge in cognitive conditions in the aging population across numerous countries. In Europe, this demographic transformation not only heralds major societal shifts but also carries substantial economic implications (WHO, 2007). As the prevalence of cognitive conditions increases within this aging cohort, the spotlight is cast on therapeutic adherence, a critical yet elusive aspect of managing chronic diseases in older adults. The implications of poor adherence reverberate across adverse health outcomes, diminished quality of life, and an alarming escalation in healthcare expenditure (Cutler et al., 2006)

Medication adherence, referring to the level of participation in terms of individuals taking medications as prescribed, is recognized as a public health problem, especially important in the treatment of chronic diseases. Older people are more likely to have concomitant chronic diseases, increasing the number of medications they take, which is a key risk factor for non-adherence. After half a century of adherence research and increased knowledge about the more than 200 factors known to influence adherence, adherence rates remain relatively unchanged (Vrijens et al., 2012; Conn and Ruppar, 2017; Ellis et al., 2023). Thus, although rates of adherence in clinical trials may be high (70%–90%), in clinical practices, they vary between 10% and 40% (WHO, 2015; Bhattarai et al., 2020). Medication adherence is essential for people to receive the full therapeutic benefits of prescribed medications, and its lack is associated with considerable morbidity and mortality. While patient behavior is important in medication non-adherence, medication adherence and its improvement are the result of complex systems that include not only individuals but also healthcare settings, healthcare policies, and healthcare professionals (Ellis et al., 2023). Effectively managing therapeutic adherence in individuals with mild cognitive impairment (MCI) and dementia is a complex task crucial for optimizing patient outcomes. In clinical practice, various methods are employed to assess adherence, each posing its unique set of challenges and opportunities.

Common methods employed to measure adherence, such as patient diaries, pill counts, and the analysis of computerized pharmacy records, bring both utility and limitations to the forefront (Osterberg and Blaschke, 2005). Pill count, while a straightforward approach, is constrained to oral medications and merely confirms the removal of the correct number of pills, offering no insights into ingestion, dosage, or frequency (Cutler et al., 2006). Simultaneously, the analysis of pharmacy records sheds light on refill patterns but remains blind to the actual ingestion or pattern of use. In the clinical setting, reliance on any single method of assessment proves potentially misleading. Therefore, the imperative emerges: it is crucial to determine the magnitude of non-adherence as the initial step toward developing targeted strategies to correct these behaviors.

Direct patient interviews and caregiver reports emerge as commonly used methods to gauge adherence. These approaches provide valuable subjective insights into medication adherence, offering a firsthand account of the patient’s experience. However, these methods may be limited by recall bias and the cognitive capacity of the patient, thus introducing potential inaccuracies in the assessment (Clifford et al., 2006). These questionnaires, adapted and validated for the Spanish population, are commonly used for chronic conditions such as hypertension and hyperlipidemia. The Morisky–Green test (MGT) measures the attitude toward treatment, while the Batalla test (BT) provides valuable information about the patients’ understanding of their illness, adding another layer to the multifaceted landscape of adherence assessment (Batalla et al., 1984; Morisky et al., 1986). In addition to interviews and caregiver reports, observing the use of practical tools like pill organizers or blister packs can offer indirect evidence of adherence behavior. These visual cues provide clinicians with tangible information about the patient’s ability to follow prescribed medication regimens and may offer insights into routine adherence patterns (Steiner and Prochazka, 1997).

Navigating the challenges of diagnosing therapeutic adherence in individuals with cognitive impairment demands a multifaceted approach. By acknowledging the limitations of subjective measures and exploring indirect indicators, clinicians can work toward a more comprehensive understanding of adherence behaviors in this unique patient population.

The objective of this study was to assess the diagnostic validity to estimate the prevalence of non-adherence in patients with MCI and dementia using two self-reported methods (SRMs) that could be useful and easy in clinical practice, considering the pill count as the reference method (RM).

## 2 Materials and methods

### 2.1 Study design

The cohort study was nested in an international multicenter randomized controlled trial SMART4MD: NCT03325699. The SMART4MD trial was approved by the Malaga Provincial Ethical Committee (30/06/2016). The protocol of the study was broadly described in a previous article (Anderberg et al., 2019).

We used the CONSORT reporting guidelines (Schulz et al., 2010).

### 2.2 Setting, participants, recruitment, and follow-up

A total of 387 patients with MCI or mild dementia were chosen, using a non-random consecutive sampling method for the SMART4MD trial, from 8 primary care centers (PCCs) and memory unit in Málaga, Spain.

The inclusion criteria were as follows: a score of 20–28 points on the Mini-Mental State Examination (MMSE) whether or not a diagnosed neurodegenerative disease is present; a professional assessment of the patient’s own experience of memory problems over a substantial period of time (more than 6 months); older than 55 years; taking prescribed medication; and are in charge of their own medication use. The exclusion criteria were as follows: a terminal illness with less than 3 years of expected survival; score above 11 on the Geriatric Depression Scale (GDS-15) (Yesavage et al., 1982a); or have another known significant cause of disease as an explanation for cognitive impairment such as abuse and other psychiatric diagnoses such as bipolar disorders, schizophrenia, and developmental disorders. These criteria were all ascertained from the patient’s clinical record.

Participants were identified from a cohort of people with cognitive impairment that has been present for more than 6 months and who met all the study eligibility criteria. Participants were under primary care services and secondary care services, such as those who are being followed up in memory clinics, outpatient clinics, day hospitals or other components of specialist mental healthcare, geriatric medicine, and neurology services. Participants were also identified from patient databases such as those integrated in the center networks. The identification process consisted of screening using information gathered from medical notes, clinic records, and/or clinical consultations for initial eligibility based on inclusion criteria.

After a brief explanation of the study design and research goals, participants were invited to participate in the study, and an appointment with the researchers was scheduled. The participants were provided with all the information they need to make an informed decision via a participant information sheet. They were given a cooling-off period of at least 24 h between informally agreeing to participate in the study and being invited to formally consent in a meeting with the research team.

At the first visit, the researcher explained the study in detail and answered any questions the patient or caregiver may have. The patient’s eligibility was confirmed, and their ability to consent was assessed. Once consent was officially given by signing an informed consent form by all parties, the subject was randomized into either the intervention or the control group for the SMART4MD trial, and a baseline visit was carried out, where all the variables were measured (this included the assessment of the treatment adherence, AT).

An 18-month follow-up was conducted after the initial visit: visit 0 (baseline), visit 1 (at 6 months), visit 2 (at 12 months), and visit 3 (at 18 months). In all visits, adherence by pill counts and self-reported adherence methods were measured.

### 2.3 Outcomes

#### 2.3.1 Treatment adherence

Adherence to a medication regimen is generally defined as “the extent to which a person’s behavior—taking medication, following a diet, and/or executing lifestyle changes—corresponds with agreed recommendations from a healthcare provider” (Lehane and McCarthy, 2009). Adherence was measured by the dose/pill count as a RM, alongside self-reported adherence methods to test the diagnostic validity.

Pill count is the number of pills or doses taken divided by the number of pills or doses prescribed, multiplied by 100 (expressed as a percentage) (Brian Haynes et al., 1980; Hansen et al., 2009). Sackett et al. (1975) suggested that good adherence is considered when the result of counting is between 80% (20% loss of doses/pills) and 110% (the patient consumes 10% more doses/pills) of doses/pills prescribed. This cutoff point was selected for consistency with other studies (Hansen et al., 2009).

Due to the polypharmacy presented in the sample, a maximum of two drugs for each participant were selected to measure the adherence. These drugs were selected following the prevalence of illness and comorbidity. In our case, medications for MCI or dementia were the most common, excluding dietary supplements, followed by hypertension and diabetes mellitus*.*

#### 2.3.2 Self-reported adherence methods

Two SRMs were selected to evaluate treatment adherence: the MGT (Hansen et al., 2009) and the BT (Batalla et al., 1984). These questionnaires that assess adherence are normally used for chronic conditions and have been adapted and validated for the Spanish population for conditions such as hypertension and hyperlipidemia (Piñeiro et al., 1997; Pineiro et al., 1997).Furthermore, the MGT is used in the Andalusian Health Service as a screening test for adherence for some chronic conditions.

#### 2.3.3 Morisky–Green test

We measured the attitude toward treatment using the MGT (Morisky et al., 1986):

(1) Do you ever forget to take your medication?
(2) Are you careless at times about taking your medication?
(3) When you feel better, do you sometimes stop taking your medication?
(4) Sometimes, if you feel worse when you take the medication, do you stop taking it?

We considered good adherence when all four questions were answered suitably.

#### 2.3.4 Batalla test

The BT provides information about the patients’ understanding of their illness (Batalla et al., 1984). The questions, adapted to the condition, used in this study were as follows:

(1) Is MCI or dementia a lifelong disease?
(2) Can you control this disease with medication or cognitive exercises?
(3) Mention one or more organs that can get damaged by your condition.

We considered good adherence when the patient answered these three questions suitably.

#### 2.3.5 Co-variables

The co-variables included sociodemographic variables (age, sex, civil status, and educational level) and clinical variables (smoking habits, number of cigarettes, treatment, and comorbidity).

Cognitive function was measured by the MMSE (Folstein et al., 1975). It is also used to estimate the severity and progression of cognitive impairment and to follow the course of cognitive changes in an individual over time. To be included in the trial, individuals must score between 20 and 28 points on the scale. The use of an MMSE cutoff value of 28 is not common and has some risks but has been used in other studies (Doody et al., 2009). O’Bryant et al. (2008) showed that an MMSE cutoff score of 28 provided the best sensitivity and specificity for detecting mild dementia in a population with self-reported memory complaints. Medical history of persons with MCI includes family antecedents such as Alzheimer’s disease, Parkinson’s disease, other dementing illness, diagnosis of dementia, type of dementia, if they have undergone a magnetic resonance imaging scan, and if they are using any pharmacological treatment for their dementia.

The GDS-15 (Yesavage et al., 1982a) was used as an exclusion criterion to screen for depression. Participants scoring above 11 on the GDS will be excluded. The GDS is commonly used as a routine part of a comprehensive geriatric assessment. The grid sets a range of 0–4 as “normal,” 5–8 as “mildly depressed,” 9–11 as “moderately depressed,” and 12–15 as “severely depressed.”

The health-related quality of life (QoL) was measured using the total score of the QoL-AD questionnaire (Logsdon et al., 2002; Thorgrimsen et al., 2003; Logsdon et al., 2002; Logsdon and Gibbons, 1999) and EuroQoL-5D. The QoL-AD questionnaire is a 13-item measure, which has been specifically designed to measure the QoL in individuals with dementia from the perspective of both the patient and the informal carer. It includes questions related to the interpersonal, environmental, functional, physical, and psychological status of the person with dementia, and thus, it is a global measure for QoL. QoL-AD will be assessed via an interview with the patient and via self-completion by informal carers. The EuroQoL-5D questionnaire is a self-completion questionnaire that consists of 5 questions plus a scale where the participant rates their health state on a scale of 0–100. EQ-5D has been shown to correlate well with QoL-AD, indicating that the two measures are compatible and can be used side by side (Thorgrimsen et al., 2003).

### 2.4 Statistical analysis

A descriptive analysis of all the study variables was conducted, calculating the mean, median, standard deviation, total frequency, and relative frequency of each category; 95% confidence intervals were calculated for the means and proportions.

We considered the dose/pill count to be the RM for assessing adherence. We performed two types of analytical strategies (Bautista Cabello Lopez and Pozo Rodríguez, 1997) to evaluate their validity to diagnose adherence: 1) open comparison to explore the existence of a statistical association between each self-reported questionnaire and the RM using the chi-squared test and 2) hierarchy comparison in which we assumed that the RM is the best method to assess non-therapeutic adherence. We then calculated the kappa value, k (as a measure of agreement between the reference method and each self-reported test), the basic diagnostic descriptors (sensitivity and specificity), and their combination (likelihood ratio: PPV and PPN) for each of the SRMs. To achieve this, we elaborated 2 × 2 tables and calculated the following indicators of diagnostic validity for each test: sensitivity = true positive/(true positive + false negative); specificity = true negative/(true negative + false positive); positive likelihood ratio = sensitivity/(1-specificity); and negative likelihood ratio = (1-sensitivity)/specificity.

A 5% significance level (α = 0.05) and the SPSS statistical package, version 25.0, were used to run the analysis described.

## 3 Results

### 3.1 Baseline characteristics

The sample consisted of 387 patients with MCI or mild dementia, of which 59.5% were female, with a mean age of 73.29 years (95% CI, 72.54–74.04), with a low educational level (71.1% with elementary school-level education). At the time of the study, 37.5% were ex-smokers, and 57.3% had never smoked (Table 1). HTA (54.4%), osteoarticular pathology (35.9%), and DM (24.5%) were the more prevalent comorbidities.

**TABLE 1:** Sociodemographic and clinical profile.

Number of subjects	387
Gender % (n)	
Male	40.5% (156)
Female	59.5% (229)
Age (mean, 95% CI)	73.29 (95% CI, 72.54–74.04)
Education level % (n)	
Elementary school	71.7% (276)
Secondary school	18.4% (71)
Higher education	8.8% (34)
Civil status % (n)	
Unmarried	4.2% (16)
Married	64.2% (247)
Common law partner	1.3% (5)
Divorced	3.6% (14)
Widowed	26.5% (102)
Living arrangement % (n)	
Single	20.8% (80)
Spouse/common law	58.2% (224)
Children	15.3% (59)
Other	5.5% (21)
Smoking habit % (n)	
Non-smokers	57.3% (217)
Smokers	5.3% (20)
Ex-smokers	37.5% (142)
MMSE (mean, 95% CI)	25.57 (95% CI, 25.34–25.8)
GDS (mean, 95% CI)	3.29 (95% CI, 2.99–3.6)

CI, confidence interval; MMSE, Mini-Mental State Examination; GDS, Geriatric Depression Scale.

Cognitive status: The MMSE mean score was 25.57 (95% CI, 25.34–25.8), and the GDS mean score was 3.29 (95% CI, 2.99–3.6). Among the participants, 29.2% had family antecedents of dementia, and 61% were in their parents. Of these, 30.9% had Alzheimer’s disease, 38.3% did not know the kind of dementia, 5.7% had vascular dementia, 2.3% had dementia with Lewy bodies, and 1.1% had frontotemporal dementia.

Drug therapy: Among the participants, 39.5% had medication for dementia, and the rest had prescription at least for another chronic condition, i.e., 40.2% for HTA and 20.3% for DM. These drugs were considered to measure the adherence in the baseline and in the follow-up visits. Regarding medication for dementia, 47.5% of the participants with a prescription had acetylcholinesterase inhibitors, 25.4% had antidepressants, 12.4% had memantine hydrochloride, 4% had antipsychotics, and 12.4% used another treatment for cognitive impairment or dementia.

Quality of life: The total QoL-AD score was 33.96 (95% CI, 33.32–34.6). For EuroQoL-5D, the subjects reported no problem with mobility (69.4%), self-care (91.9%), and daily activities (80.5%). Furthermore, 46.5% reported no pain or discomfort, and 43.4% had moderate pain or discomfort. Regarding anxiety/depression, 64.7% reported no symptoms, 30% felt moderately anxious or depressed, and 4.4% were extremely anxious or depressed.

### 3.2 Follow-up

V2: A total of 283 patients (73.3%) of the 386 included in the study attended the first follow-up visit 6 months after inclusion. A total of 103 patients did not attend this visit, i.e., 22.34%. A total of 23 patients were dropped out for this visit because they were unable to attend the visit (19) or were unreachable (4). A total of 80 participants (20.72%) were dropped out for the study because they did not want to continue in the study, were excluded because they did not meet the inclusion criteria, or were deceased.

V3: A total of 250 patients (64.8%) of the 386 included in the study attended the second follow-up visit 1 year after inclusion. A total of 136 patients did not attend this visit, i.e., 35.2%. A total of 28 patients were dropped out for this visit because they were not able to attend the visit (25) or were unreachable (3). The dropout rate for the study increased by 28 participants, resulting in a dropout rate of 27.9% (108), because the participants did not want to continue in the study, were excluded because they did not meet the inclusion criteria, or were deceased.

V4: A total of 223 patients (57.8%) of the 386 included in the study attended the second follow-up visit 1 year after inclusion. A total of 163 patients did not attend this visit, i.e., 42.2%. A total of 42 patients were dropped out for this visit because they were not able to attend the visit (38) or were unreachable (4). The dropout rate for the study increased by 17 participants, resulting in a dropout rate of 32.3% (125), because the participants did not want to continue in the study, were excluded because they did not meet the inclusion criteria, or were deceased.

### 3.3 Treatment adherence and diagnosis validity of the self-reported test

**FIGURE 1:**
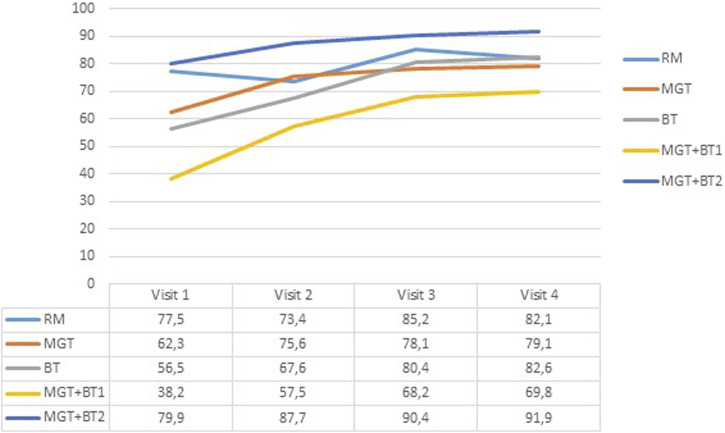
Evolution of the adherence treatment percentage after 18 months of monitoring % of patients. RM: pill count; MGT: Morisky–Green test; BT: Batalla test; MGT + BTl: adherent patients’ diagnoses using at least two methods; MGT + BT2: adherent patients’ diagnoses using the two methods.

Figure 1 shows the evolution of the treatment adherence during the study measured by the different reported methods: pill count as RMs, MGT, BT, and the combination or both (MGT + BT1 and MGT + BT2).

In the baseline, non-adherence prevalence using the RM was 22.5%. The non-adherence prevalence for the self-reported adherence methods was 37.7% for the MGT and 43.5% for the BT. The MGT detected 39 of the 67 patients classified as non-adherent using the RM, while the BT found 42. Considering both tests together and when the subject is classified as non-adherent for both tests (MGT + BT2) in the baseline, 24 non-adherent patients were detected. Finally, when we consider both tests together and when the subject is classified as non-adherent for at least one of the tests (MGT + BT1), 54 non-adherent patients were detected.

The chi-squared test showed a significant association between the RM and the SRMs (Table 2). The measure of agreement by kappa (k) between the MGT and the RM was 0.215 (*p* ≤ 0.001), for the BT, k = 0.25 (*p* ≤ 0.001), and when we considered both tests together, k = 0.262 (*p* ≤ 0.001) for MGT + BT2 and k = 0.214 (*p* ≤ 0.001) for MGT + BT1.

**TABLE 2:** Open comparison of adherence prevalence between the self-reported methods and the reference method using the chi-squared test.

	Baseline	6 months	12 months	18 months
Pill count	77.5% (77.08–77.9)	73.4% (72.9–73.9)	85.2% (84.85–85.55)	82.1% (81.7–82.48)
Morisky–Green test (MGT)	62.3% (61.8–62.78) *p* ≤ 0.001	75.6% (75.17–76.07) *p* = 0.006	78.1% (77.69–78.51) *p* ≤ 0.001	79.7% (79.3–80.1) *p* ≤ 0.001
Batalla test	56.5% (56–57) *p* ≤ 0.001	67.6% (67.13–68) *p* ≤ 0.001	80.4% (80–80.8) *p* = 0.002	82.6% (82.2–82.9) *p* ≤ 0.001
MGT + BT	79.9% (79.5–80.3) *p* ≤ 0.001	87.8% (87.47–88.13) *p* = 0.039	90.4% (90.1–90.7) *p* = 0.001	91.9% (91.6–92.1) *p* = 0.001

In visit 1, 6 months after inclusion, the non-adherence prevalence using the RM was 26.2%. The non-adherence prevalence for the self-reported adherence methods was 24.2% for the MGT and 32.4% for the BT. The MGT detected 24 of the 61 patients classified as non-adherent using the RM, while the BT found 33. Considering both tests together and when the subject is classified as non-adherent for both tests (MGT + BT2), 12 non-adherent patients were detected. Finally, when we consider both tests together and when the subject is classified as non-adherent for at least one of the tests (MGT + BT1), 40 non-adherent patients were detected.

The chi-squared test showed a significant association between the RM and the SRMs (Table 2). The measure of agreement by kappa (k) between the MGT and the RM was 0.2 (*p* ≤ 0.001), for the BT, k = 0.344 (*p* ≤ 0.001), and when we considered both tests together, k = 0.153 (*p* = 0.012) for MGT + BT2 and k = 0.337 (*p* ≤ 0.001) for MGT + BT1.

In visit 2, 12 months after inclusion, the non-adherence prevalence using the RM was 14.8%. The non-adherence prevalence for the self-reported adherence methods was 21.9% for the MGT and 19.6% for the BT. The MGT detected 17 of the 31 patients classified as non-adherent using the RM, while the BT found 12. Considering both tests together and when the subject is classified as non-adherent for both tests (MGT + BT2), eight non-adherent patients were detected. Finally, when we consider both tests together and when the subject is classified as non-adherent for at least one of the tests (MGT + BT1), 20 non-adherent patients were detected.

The chi-squared test showed a significant association between the RM and the SRMs (Table 2). The measure of agreement by kappa (k) between the MGT and the RM was 0.321 (*p* ≤ 0.001), for the BT, k = 0.22 (*p* ≤ 0.001), and when we considered both tests together, k = 0.236 (*p* = 0.001) for MGT + BT2 and k = 0.282 (*p* ≤ 0.001) for MGT + BT1.

In visit 3, 18 months after inclusion, the non-adherence prevalence using the RM was 17.9%. The non-adherence prevalence for the self-reported adherence methods was 20.3% for the MGT and 17.4% for the BT. The MGT detected 18 of the 33 patients classified as non-adherent using the RM, while the BT found 16. Considering both tests together and when the subject is classified as non-adherent for both tests (MGT + BT2), eight non-adherent patients were detected. Finally, when we consider both tests together and when the subject is classified as non-adherent for at least one of the tests (MGT + BT1), 25 non-adherent patients were detected.

The chi-squared test showed a significant association between the RM and the SRMs (Table 2). The measure of agreement by kappa (k) between the MGT and the RM was 0.399 (*p* ≤ 0.001), for the BT, k = 0.41 (*p* ≤ 0.001), and when we considered both tests together, k = 0.265 (*p* ≤ 0.001) for MGT + BT2 and k = 0.474 (*p* ≤ 0.001) for MGT + BT1.

The diagnostic validity of the SRM is shown in Table 3.

**TABLE 3:** Diagnostic validity of self-reported methods to detect non-adherent patients with the prescribed treatment.

	Baseline	6 months	12 months	18 months
Non-adherence (reference method)	22.5	26.2	14.8	17.9
Morisky–Green test				
Non-adherence	37.7	24.2	21.9	20.3
Sensitivity	58.2% (46.4–70)	39.3% (27–51.6)	54.8% (37.3–72.3)	54.5% (37.5–71.5)
Specificity	68.4% (62.4–74.4)	80.7% (74.7–86.6)	83.7% (78.2–89.1)	87.2% (81.8–92.6)
Positive predictive value	35%	42%	37%	48%
Negative predictive value	85%	78%	91%	89%
Positive likelihood ratio	1.84	1.96	3.55	4.15
Negative likelihood ratio	0.6	0.76	0.53	0.53
Batalla test				
Non-adherence	43.5	32.4	19.6	17.4
Sensitivity	70% (81.5–58.4)	61.1% (48.1–74.1)	40% (22.4–57.5)	50% (32.6–67.3)
Specificity	64.25% (56–72.5)	77.3% (70.8–83.7)	84.4% (79–90)	90% (85–95)
Positive predictive value	35%	47%	31.5%	53%
Negative predictive value	88%	85%	88%	89%
Positive likelihood ratio	1.95	2.77	2.5	5
Negative likelihood ratio	0.46	0.5	0.71	0.55
MGT + BT1 (non-adherent at least by one of the two methods)				
Non-adherence	30.2	31.8	42.5	61.8
Sensitivity	90% (82.4–97.5)	74% (62.3–85.7)	66.6% (50–83.5)	78.1% (63.8–92.4)
Specificity	46.1% (39.5–52.7)	68.1% (61–75.2)	74.2% (67.6–80.8)	80.7% (74.1–87.2)
Positive predictive value	31%	43%	66%	48%
Negative predictive value	94%	88%	92%	94%
Positive likelihood ratio	1.66	2.31	2.54	3.9
Negative likelihood ratio	0.22	0.38	0.46	0.275
MGT + BT2 (non-adherent by the two methods)				
Non-adherence	20.1	12.3	9.6	8.1
Sensitivity	40% (27.6–52.4)	22.2% (11.1–33.3)	26.6% (0.11–0.42)	25% (10–40)
Specificity	85.5% (80–90)	90.7% (86.3–95.2)	93.4% (89.6–97.1)	95.7% (92.3–99)
Positive predictive value	42%	44%	42%	57%
Negative predictive value	84%	77%	87%	84%
Positive likelihood ratio	2.66	2.2	3.71	6.25
Negative likelihood ratio	0.7	0.45	0.79	0.78

## 4 Discussion

The analysis found that the diagnostic validity of self-reported questionnaires to measure non-adherence administered independently in patients with MCI or early stages of dementia is low, especially when non-adherence is infrequent. Using the two questionnaires studied together and considering a patient non-adherent if deemed so by at least one of the two questionnaires is an acceptable way to estimate non-adherence. Additionally, it imposes minimal burden on clinicians and/or researchers, as well as on patients, since they are brief tests that can be administered at the same time. The BT yielded slightly better results than the MGT, and therefore, we recommend its use if administering both is not feasible.

MCI has been associated with problems to adhere to the multiple medication regimens frequently followed by older adults (Kröger et al., 2017). Medication adherence is fundamental to adequately treat conditions that could negatively impact cognitive impairment and dementia, such as diabetes and hypertension. Likewise, non-adherence to medication has been associated with a worse prognosis of cognitive deficit (Gard, 2010). Therefore, having a reliable, valid, and simple method to evaluate adherence in this population is essential to evaluate and develop interventions and achieve improvements that result in a better prognosis and quality of life in this population.

When we considered both tests, both tests classify the patient as non-adherent, observing a considerable increase in the specificity with a reduction in sensitivity. These values match those of other trials for chronic diseases, in which the specificity overcomes sensitivity (Pineiro et al., 1997; Pineiro et al., 1997). In our case, this means that we would classify correctly adherent subjects (true negative) because sensitivity is low, and specificity is high. In clinical practice, this is very useful because when both tests are used with a patient, and they are classified as adherent, it indicates that they are well diagnosed. If we consider the likelihood ratio to detect non-adherent patients, we see that both tests identify a patient as non-adherent with the scheduled inhaled treatment, and it is nearly 6-fold more likely to be a true positive value.

It has been observed that participants’ adherence throughout the study increases, which may be explained by the Hawthorne effect (Sedgwick and Greenwood, 2015), wherein the continuous assessment of adherence throughout the study may modify the negative pattern of medication intake and increase the likelihood of following pharmacological guidelines correctly. On the other hand, being aware of a future evaluation also increases motivation and improves performance. Furthermore, the improvement in adherence over visits may also be attributed to selective experimental mortality (Jurs and Glass, 1971), i.e., fewer trial dropouts among those adhering to the treatment.

Despite initial concerns about the reliability of questionnaires for estimating pharmacological treatment adherence in patients with cognitive impairment or dementia (Arlt et al., 2012), given memory problems (Luck et al., 2007) and difficulties in monitoring behavior (Volicer, 2018), the results are comparable or even better than those found in similar studies with samples of patients without cognitive impairment (Barnestein-Fonseca et al., 2011). Another noteworthy point is that the BT, although inferring non-adherence in a less direct manner, seems to have obtained better results, which could be attributed to social desirability (Stirratt et al., 2015), affecting the questionnaire validity when directly inquiring about adherence. The use of two forms of measuring adherence, one more direct and the other more indirect, might explain why using both and considering non-adherence with only one of the methods can be an acceptable way to estimate non-adherence due to the high specificity of these tests. In general, it has been frequently observed that adherence estimates from self-reported questionnaires often do not align with other methods (Garber et al., 2004).

### 4.1 Limitations

The obtained results have several limitations. First, pill counting involves biases and is not a perfect method. The most well-known bias is that it tends to overestimate adherence, possibly explaining the differences in the percentages of non-adherent individuals obtained through the two methods, with more non-adherent individuals when questionnaires are used. Additionally, the study is based on a clinical trial, representing a specific population with high levels of adherence, especially among those who complete follow-ups, thus limiting external validity. Furthermore, the study population comes from only one of the three centers participating in the clinical trial, thus limiting the generalizability of the results.

## 5 Conclusion

The studied self-reported tests used collectively can provide valuable information regarding adherence in older individuals with MCI, as extensively demonstrated in this and other medical conditions (Stirratt et al., 2015). However, they exhibit low sensitivity, which must be considered when used, and is related to the challenge of accurately measuring treatment adherence. On the other hand, the specificity is high, and in daily clinical practice, this is very useful because when both tests are used with a patient and he or she is classified as adherent, then this is the case.

Although the methods used to measure adherence are not perfect, it is better to use them in a homogeneous and structured manner rather than not to take them into account. The dose/pill count could be chosen in clinical practice, even though we know that it overestimates adherence. An alternative to the pill count is an SRM, but the diagnostic validity of the two tests performed independently is low. Nevertheless, when they are considered together, they have a higher potential to detect patients with non-adherence to therapeutic regimens and at a low cost and in a reliable way in daily clinical practice.

In the context of aging societies and the promotion of dementia-friendly societies, this work contributes by shedding light on the importance of medication adherence in managing cognitive decline in MCI and early-stage dementia patients, thereby potentially improving their quality of life and overall wellbeing.

## Data Availability

The raw data supporting the conclusion of this article will be made available by the authors, without undue reservation.
